# Targeting the GLP-1/GLP-1R axis to treat osteoarthritis: A new opportunity?

**DOI:** 10.1016/j.jot.2022.02.001

**Published:** 2022-02-25

**Authors:** C. Meurot, C. Jacques, C. Martin, L. Sudre, J. Breton, R. Rattenbach, K. Bismuth, F. Berenbaum

**Affiliations:** a 4P-Pharma, Lille, France; b Sorbonne University, INSERM UMRS_938 and Labex Transimmunom, CDR St-Antoine Paris, Paris, France; c 4Moving Biotech, Lille, France; d APHP, Sorbonne University, Rheumatology Department, INSERM UMRS_938, CDR St-Antoine Paris, Paris, France

**Keywords:** Osteoarthritis, Glucagon-like peptide-1 (GLP-1), Glucagon-like peptide-1 receptor (GLP-1R), Liraglutide, Cartilage, Synovial tissue

## Abstract

Osteoarthritis (OA) is a degenerative joint disease affecting millions of people worldwide. In OA, chondrocytes, synovial cells and other joint cells become activated when exposed to an abnormal environment, including mechanical stress, inflammatory cytokines or disorganization of matrix proteins. Several analogues of the hormones called incretins have been developed and are used notably for treating type 2 diabetes mellitus. Data has accumulated to suggest that incretinomimetics, which bind to the glucagon-like peptide-1 receptor (GLP-1R), have beneficial pleiotropic effects such as immunomodulation, anti-inflammation and neuronal protection. Thus, because of their anti-inflammatory properties, GLP-1–based therapies could benefit OA patients. This review focuses on the GLP-1R pathway, molecular mechanisms and phenotypes related to OA pathogenesis.

**The translational potential of this article:**

The search for new therapeutic targets to treat people suffering from OA remains urgent as there is currently no disease-modifyingtherapy available for this disease. This review discusses how GLP-1 analogues could be potential DMOADs for treating OA thanks to their anti-inflammatory, immunoregulatory and differentiation properties.

## 1 Introduction

Osteoarthritis (OA) is the most common degenerative joint condition and the leading cause of disability, affecting over 300 million individuals worldwide [1,2]. In the absence of disease-modifying treatments, the US Food and Drug Administration (FDA) has characterized OA as a serious disease [3]. OA affects the joints, including knees, hands, hips and spine, and is the leading cause of impaired mobility in older people [1]. Risk factors for knee and hip OA include age, sex, obesity and cardiometabolic factors, all of which are central features of metabolic dysfunction. However, other risk factors for OA include trauma, sports activities and anatomical abnormalities. The overlap between OA and metabolic dysfunction prompted scientists to propose that OA might be a component of the metabolic syndrome [4,5]. Traumatic joint injury is another risk factor for the development of OA (post-traumatic osteoarthritis [PTOA]), associated with earlier disease onset than age-related OA and more readily affecting younger patients [6]. The mechanical injury is accompanied by release of cytokines such as interleukin 1 or 6 (IL-1 or 6), with both acute and long-term deleterious cellular effects: necrosis, apoptosis, autophagy and/or senescence [6]. In addition, mechanical activation of chondrocytes, which occurs during injury, activates extracellular matrix degradative enzymes including matrix metalloproteinases (MMPs) and a disintegrin and metalloproteinase with thrombospondin motifs (ADAMTS) [6]. Inflammation has a fundamental role in OA initiation and development. The inflammation found in OA is low grade and is induced and sustained by the innate immune response, metabolic syndrome and inflammaging [7].

Type 2 diabetes mellitus (T2DM) and OA are common diseases that are frequently associated. The prevalence of OA in patients with T2DM is 52%, whereas the prevalence in the general population is 27% [8]. The well-established association between OA and diabetes is explained at least in part by hyperglycemia, which, at the level of joint tissues, causes cellular and tissue toxicity [9]. In the past century, the discovery and characterization of the incretins, a family of gastrointestinal hormones that stimulate insulin production, has enabled the development of new therapies for treating T2DM, a chronic disease characterized by elevated blood glucose level caused by insulin resistance [10,11]. Glucagon-like peptide-1 (GLP-1) and glucose-dependent insulinotropic polypeptide (GIP) are the two main incretin hormones known to stimulate glucose-dependent insulin secretion by pancreatic β cells. However, the rapid degradation of native human GLP-1 by dipeptidyl peptidase 4 (DPP-4) has limited its clinical application and prompted the development of GLP-1 analogues (*e.g.,* liraglutide, exenatide, semaglutide, lixisenatide) that are resistant to DPP-4 cleavage, which are now used for treating T2DM [12]. Beyond its insulinotropic effects, GLP-1 has beneficial physiological activities in the brain [13], heart [14], and lung [15], sustained notably by strong anti-inflammatory activities [10,16]. GLP-1 binds to GLP-1 receptor (GLP-1R), a G-coupled receptor readily found in pancreatic β cells, intestine and central nervous system and moderately expressed in blood vessels, pancreatic alpha cells, peripheral nervous system, lungs, heart, kidneys and joints [7]. Taken together, with the overlap between OA risk factors and the metabolic syndrome, the demonstrated anti-inflammatory activities of GLP-1 in several tissues [10] and the expression of GLP-1R in joint tissues, GLP-1 analogues may be good therapeutic candidates for treating OA. Drugs that have already demonstrated strong properties in decreasing low-grade systemic inflammation could also act locally at the joint level [17]. It follows that the use of incretinomimetics activating the GLP-1R pathway may be an interesting lead for treating OA.

The aim of this review was to provide a comprehensive overview of the role of the GLP-1/GLP-1R axis in the normal and pathological physiology of joint tissues. We focus on describing 1) how the dysregulation of GLP-1–mediated molecular pathways can contribute to the development of OA and 2) the promising clinical application of GLP-1 analogues as therapies for OA.

### 1.1 GLP-1 and GLP-1R

In 1932, Jean La Barre purified the glucose-lowering element from gut extracts and named it INtestine seCRETtion Insulin (incretin) [[18], [19], [20]]. The observation that oral versus intravenous glucose administration produced a stronger insulin response is at the core of the incretin effect, defined as the difference in pancreatic β-cell insulin secretory response to glucose administered orally or intravenously [21].

Incretins are a large family of metabolic hormones also called the “glucagon superfamily of peptides,” with the most-described and documented physiological effects being performed by GLP-1 (7–37) and (7–36) amide and GIP. GIP is a 42-amino acid peptide secreted from upper small intestine K cells [20] and was the first incretin hormone to be isolated in the 1970s by John Brown [19,22]. In the 1980s, Drucker et al. performed a seminal work on the sequencing and characterization of the second incretin hormone, GLP-1 [23].

GIP and GLP-1 bind to their respective receptors: the GIP receptor (GIPR) and GLP-1R to mediate their pleiotropic cellular effects [10]. Both GIPR and GLP-1R belong to the G protein-coupled receptor family. We narrowed the focus of the present review to GLP-1 incretin and its downstream signalling pathway in joint tissues.

GLP-1 is a 30- or 31-amino acid peptide derived from the prohormone convertase 1 post-translational cleavage of the pro-glucagon polypeptide. GLP-1 is produced and secreted by intestinal enteroendocrine L cells upon nutrient intake as well as in glucagon-expressing neurons in the brain [24]. The initial immature form, GLP-1 (1–37), is cleaved and undergoes amidation, producing two equipotent truncated peptides, GLP-1 (7–37) and the main biologically active GLP-1 (7–36). GLP-1 consists of two α-helices from amino acid position 13–20 and 24-35 separated by a linker region. The C-terminal helix of GLP-1 binds to the extracellular domain of GLP-1R, whereas the N-terminal region of GLP-1 interacts with the GLP-1R transmembrane domain [25]. Endogenous GLP-1 is rapidly cleaved primarily by DPP-4 as well as neutral endopeptidase 24.11 and eliminated by renal clearance, thus resulting in a short half-life of approximately 2 ​min [26]. Only 10%–15% of active GLP-1 reaches the general circulation intact, thus leading to minimal fasting plasma levels of 0–15 ​pmol/L [24]. The very short plasmatic half-life represents a major limitation for the possible use of GLP-1 in the clinical setting. However, GLP-1 mimetics have been designed to have increased binding to large blood circulating proteins such as albumin, thus providing a much longer half-life than native GLP-1 (e.g., ±13 ​h for liraglutide, commercially known as Victoza®) [27]. These GLP-1 analogues were found effective for T2DM because they prolong the incretin effect in patients [27]. The most recent incretinomimetics are exenatide (Byetta®, Bydureon®), approved in 2005 and 2012, respectively; liraglutide (Victoza®, Saxenda®), approved in 2010 and 2014 for treating T2DM and obesity, respectively; albiglutide (Tanzeum® or Eperzan®), approved in 2014; dulaglutide (Trulicity®), approved in 2014; lixisenatide (Lyxumia® or Adlyxin®), approved in 2015 and 2016, respectively; and semaglutide (Ozempic®, Rybelsus®), approved in 2017. These agents work by activating the GLP-1R rather than inhibiting the breakdown of GLP-1, as do DPP-4 inhibitors, and are considered more potent (Table 1).

**Table 1:** Comparison of selected clinical and pharmacokinetic plasma parameters of approved glucagon-like peptide-1 receptor (GLP-1R) agonist drugs used in type 2 diabetes mellitus.

GLP-1 analog	Commercial name	Year of approval (FDA/EMA)	Company	Description	Indication	Form	Half-life	Cmax	Dose and dose regimen	Informations
Albiglutide	Tanzeum® (US)	2014 (not available in France)	GlaxoSmithKline	Fused to human albumin, extended-release	Type 2 diabetes	Pen, SC injection	5–6 days	1.74 ​μg/ml	Once weekly, 30 or 50 ​mg	2nd purpose, any time
	Eperzan® (UE)									Any time
Dulaglutide	Trulicity®	2014	Lilly	Fragment crystallizable region of human IgG4, extended-release	Type 2 diabetes	Pen, SC injection	4.7 days	114 ​ng/ml	Once weekly, 0.75 or 1.5 ​mg	Any time
Exenatide	Byetta®	2005	Lilly	Synthetic Exendin-4, immediate release	Type 2 diabetes	Pen, SC injection	2.4 ​h	211 ​pg/ml	Twice daily, 5 ​μg or 10 ​μg	Before the meal
	Bydureon®	2012	Astra Zeneca	Synthetic Exendin-4, extended-release			3–5 days		Once weekly, 2 ​mg	Same day, any time
Liraglutide	Victoza®	2010	Novo Nordisk	Linked with a fatty acid, immediate release	Type 2 diabetes	Pen, SC injection,	12–13 ​h	35 ​ng/ml	Once daily, 0.6 ​mg and 1.2 or 1.8 ​mg	Fixed time
	Saxenda®	2014			Obesity or overweight related to Type 2 diabetes				Once daily, 3 ​mg	
Lixisenatide	Lyxumia® (UE)	2013	Sanofi	Exendin-4 analog, immediate-release	Type 2 diabetes	Pen, SC injection	3–4 ​h	175 ​pg/ml	Once daily, 10 ​μg then 20 ​μg	Before meal
	Adlyxin® (US)	2016							Once daily, 20 ​μg	Before the first meal of the day
Semaglutide	Ozempic®	2017	Novo Nordisk	Linked with a fatty acid, extended-release	Type 2 diabetes	Pen, SC injection	7 days	41.2 ​ng/ml	Once weekly, 0.25 ​mg then 0.5 ​mg	Any time
	Rybelsus®			Coformulation with an absorption enhancer protects, extended-release		Tablet, Oral	7 days	26.3–60 ​ng/ml	Once daily, 3 ​mg (1 month), 7 ​mg (1 month), 14 ​mg	Empty stomac, any time

### 1.2 Multiple biological activities of GLP-1

GLP-1 analogues stimulate glucose-dependent insulin secretion, dampen inappropriately high glucagon secretion, slow gastric emptying and reduce food intake [28]. GLP-1 acts within the gut–brain axis; indeed, although it reaches the brain *via* endocrine mechanisms, it also acts locally by activating GLP-1R present on the dendritic terminals of the vagus nerve that innervate the gut, thus mediating the inhibition of food intake as well as relaying the satiety signal to the brain. In the brain, GLP-1R expression is found in several nuclei in the hypothalamus that are involved in controlling food intake and body weight [29]. Recently, the combination of GLP-1–mediated signalling pathways and the adipocyte hormone leptin has raised interest. Indeed, leptin could be an important biological signal by which GLP-1 interacts additively or synergistically to reduce food intake and body weight. This leptin–GLP-1 action may be mediated in part by common and complementary intracellular signalling pathways (phosphorylated signal transducer and activator of transcription 3 [STAT3], PTP1B) [29]. Beyond their hypoglycemic and metabolic effects, GLP-1–based therapies have demonstrated anti-inflammatory effects, with *in vitro* and *in vivo* accumulating evidence demonstrating that GLP-1 reduces levels of inflammatory markers [[30], [31], [32]]. GLP-1–based therapies downregulate pro-inflammatory responses in inflammatory chronic-related diseases such as diabetes, vascular diseases, neurodegenerative brain disorders, non-alcoholic steatohepatitis, nephropathy [33] and OA [17,[34], [35], [36]].

The protective cardiovascular effects of a GLP-1 analogue was demonstrated in a mouse model of non-diabetic angiotensin II-induced arterial hypertension. In this model, liraglutide showed vascular protection by preventing the uncoupling of inducible nitric oxide synthase (iNOS) and reduced immune cell infiltration into the heart by decreasing levels of pro-inflammatory signals, namely, NF-κB, TNF, and IL-1β [37].

The anti–neuro-inflammatory activity of GLP-1 analogues has been investigated in animal models of neurodegenerative diseases, with encouraging results [38]. Liraglutide (NCT01843075 and NCT02953665), lixisenatide (NCT03439943), semaglutide (NCT04777396 and NCT03659682) and exenatide (NCT01255163 and NCT01971242) are being tested in clinical trials for Alzheimer's and Parkinson's diseases. These potential future treatments have improved coordination and motor activity in patients [39,40].

### 1.3 GLP-1/GLP-1R functions in different knee joint tissues

**Fig. 1:**
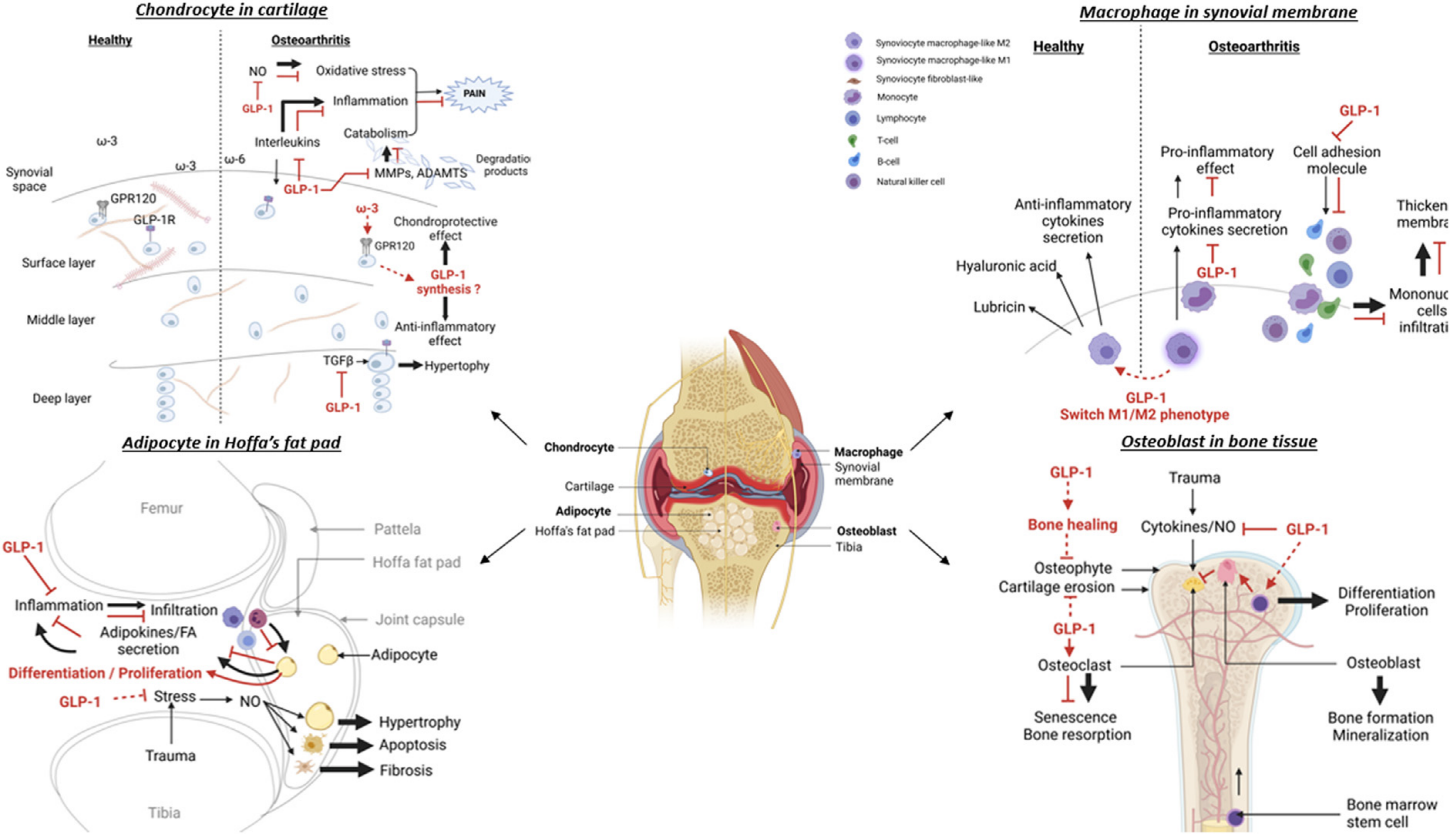
Proposed targets of glucagon-like peptide-1 (GLP-1) in joint tissues. Anatomical illustration of osteoarthritis knee joint structure including the main contributing tissues and their interactions. The cell-specific roles and molecular effects of GLP-1 in the GLP-1 receptor (GLP-1R)-dependent pathway may help counteract the pathogenesis of osteoarthritis (OA) in cartilage, synovial membrane, Hoffa's fat pad and bone tissue. GLP-1R is expressed in chondrocytes, macrophages, adipocytes and the osteocyte surface. G protein-coupled receptor 120 (GPR-120) is also expressed on the chondrocyte surface. GLP-1 or GLP-1 analogues mediate their effects by binding to the GLP-1R. The main effects lead to inhibition of cytokine secretion into the synovial fluid, thus decreasing inflammation and consequently reducing other downstream effects such as oxidative stress, pro-degradative mediator secretion, phenotype modification (hypertrophy, M1/M2 macrophage phenotype, fibrosis) or impairment/destruction of joint cells (apoptosis, senescence). GLP-1 or GLP-1 analogues can also induce anabolic mechanisms such as cell proliferation and differentiation, mineralization or healing.

**Fig. 2:**
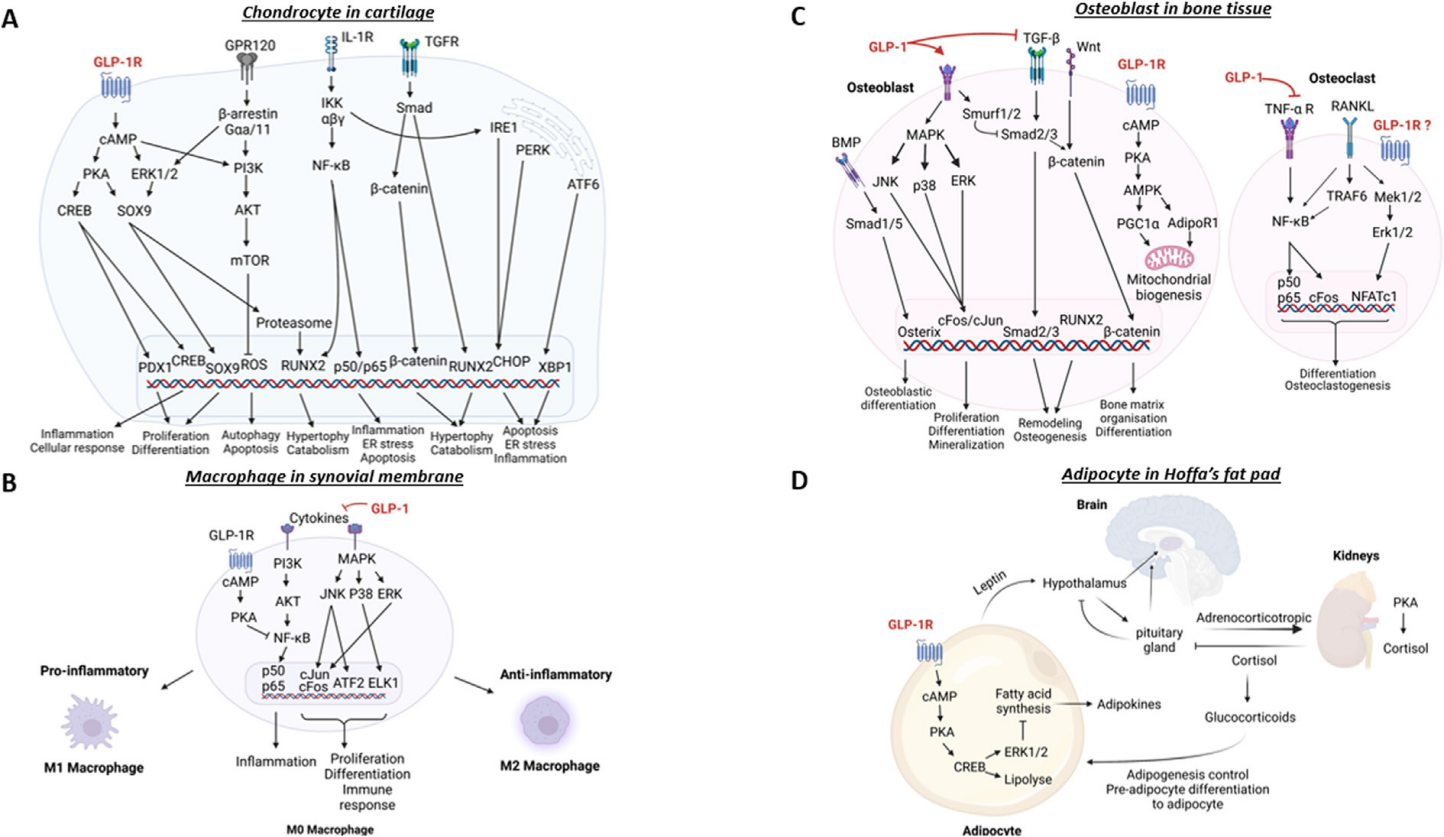
GLP-1R signalling pathways in different cell types of the joint. Proposed models of intracellular network associated with activation of GLP-1R regulating chondrocytes (A), macrophages (B), osteoblast/osteoclasts (C) and adipocytes (D). GLP-1 or GLP-1 analogues bind to GLP-1R and activate adenylyl cyclase inducing cyclic AMP (cAMP) release, which principally activates (PKA/CREB) and ERK1/2 pathways. These pathways are available to stimulate the expression of several genes involved in protective or repair effects such as SOX9 or CREB. Other physiological effects mediated by GLP-1 include lipolysis, bone metabolism, or mitochondrial biogenesis. Moreover, in chondrocytes, cAMP can indirectly activate the PI3K/Akt signalling pathway. This pathway inhibits autophagy, apoptosis as well as inflammation, endoplasmic reticulum (ER) stress and catabolism *via* inhibition of NF-κB pathway. GLP-1 can also indirectly inhibit the NF-κB pathway by inhibiting levels of cytokines present in synovial fluid owing to the GLP-1 anti-inflammatory effect. This strong anti-inflammatory activity allows GLP-1 to promote a switch of macrophage phenotype from M1 pro-inflammatory to M2 anti-inflammatory. In adipocytes, the secretion of leptin is captured by the hypothalamus and the pituitary gland, which will induce the secretion of cortisol by the kidneys and allow for better management of adipogenesis. In bone cells, GLP-1 promotes the osteoblastogenesis process.

OA is a whole-joint degenerative disease, and pathological changes seen in OA include degradation of cartilage, synovial inflammation and thickening of subchondral bone with osteophyte formation, associated with symptoms such as pain and stiffness. Changes in intra-articular fat pads and nerves can also contribute to OA development [41]. There is no direct research evidence about the therapeutic effects of GLP-1-based therapies in OA in humans, which represents a limitation of current research stage. However, pharmacological and preclinical data are gathering (Table 2). Here we introduce the existing evidence for GLP-1R expression in different cell types of the joint and a role for the GLP-1/GLP-1R couple in joint homeostasis (see ​Fig. 1). We focus on the molecular effectors of GLP-1R activation in the different joint tissues and how they interplay (see ​Fig. 2). We also present the accumulating pharmacological data for use of GLP-1 analogues as disease modifiers of OA.

• **Cartilage and GLP-1R signalling**

**Table 2:** GLP-1 functions and GLP-1R signalling pathways in osteoarthritis.

**Healthy conditions**	**Osteoarthritis conditions**	**GLP-1 analogues effects**	**Signaling pathways involved in GLP-1 effects**
Homeostasis	Inflammation Cytokines synthesis	Anti-inflammatory (10, 16,30–32, 49–50, 63)	NF-κB PKA/CREB MAPK
Cartilage synthesis Matrix production	Hypertrophic differentiation ER stress	Anabolism/chondrogenic differentiation (43) Anti-oxydative stress (16)	? ? PI3K/Akt MAPK
Cartilage degradation	Catabolism Apoptosis Decreased Autophagy Senescence	Anti-catabolic (ROS, AGEs) Prevent apoptosis (35) ? ?	PI3K/Akt MAPK PI3K/Akt AMPK ? ?
Osteogenesis	Subchondral bone remodeling Osteophytes Reduced mineralization	Proliferation/ Differentiation (62–66) Maturation (69) Migration (63) Autophagy	Erk 1/2 MAPK β-catenin RANKL Wnt/β-catenin ? TGF-β
Adipogenesis	Adipokines synthesis Fatty acid synthesis Inflammation	Proliferation/ Differentiation (75,77) Fatty acid degeneration (47) Anti-inflammatory	PKC ? NF-κB PKA/CREB Erk 1/2
Nociception	Neuro-inflammation Pain Neuronal apoptosis	Neurotrophic/ Neuroprotector (92) Anti-inflammatory (86) Improved pain sensitivity (88) (release of β-endorphin) Analgesic (85) Anti-apoptotic (90)	Erk 1/2 NF-κB PKA/CREB cAMP MAPK Erk 1/2 PI3K/Akt AMPK

Articular cartilage is a connective tissue composed of chondrocytes and an extracellular matrix of water, high-molecular-weight proteoglycans and collagen. Chondrocytes ensure the synthesis and renewal of the extracellular matrix and as such, play a crucial role in the maintenance of its homeostasis. At the cellular level, OA is the result of an imbalance between anabolic and catabolic processes. The pathogenesis of OA is driven by accumulative dysregulated pathways, including oxidative stress, autophagy, senescence, secretion of pro-inflammatory cytokines with activation of the NF-κB pathway and elevated secretion of the matrix-degrading enzymes metalloproteinases (mainly MMP-3, MMP-13) and aggrecanases (ADAMTS4, ADAMTS5).

Only very recently has the role of the GLP-1R signalling pathway in chondrocytes been explored, and it remains to be further detailed. GLP-1R was detected by immunohistochemistry in normal and OA articular chondrocytes in rat knee sections [35]. GLP-1R signalling is associated with preventing apoptosis, anti-inflammatory activity and matrix protection.

Indeed, liraglutide protected rat chondrocytes against IL-1β– or thapsigargin-induced apoptosis. Liraglutide exerts its anti-apoptotic activity by activating the PI3K/Akt signalling pathway, leading to endoplasmic reticulum stress-associated apoptosis, an increase in protein level of pro-survival B-cell lymphoma 2 and a decrease in level of pro-apoptotic cleaved caspase 3 [35]. Liraglutide's protective effect was further verified in the anterior cruciate ligament (ACL) rat model, in which sub-cutaneous injection of liraglutide 50 ​μg/kg/day for 3 or 6 weeks decreased the number of caspase 3- and CHOP-positive cells in cartilage [35].

GLP-1R activation is associated with decreased activity of the NF-κB pathway [42], which orchestrates a wide range of stress-related inflammatory responses and controls the growth, survival, and development of many cell types. In TNF-stimulated human chondrocytes [42] or thapsigargin-stimulated rat chondrocytes [35], inhibition of the NF-κB pathway resulted in less secretion of pro-inflammatory mediators such as IL-6, CCL2 [42] and TNF [35]. In primary mouse chondrocytes, treatment with liraglutide reduced the mRNA expression of *iNOS, MMP-13 and ADAMTS5*, thus leading to reduced secretion of a battery of inflammatory markers such as NO, prostaglandin E_2_, and IL-6 [17]. Using the monoiodoacetate (MIA) rat model of inflammatory OA, Que and colleagues demonstrated that GLP-1R activation triggered the protein kinase A/cAMP-response element binding protein (PKA/CREB) signalling pathway, thus reducing inflammation in cartilage [36].

GLP-1 analogues possess anti-catabolic activity, as demonstrated by reduced *MMP-3* and *MMP-13*, *ADAMTS4*, *ADAMTS5* mRNA expression in TNF-stimulated human chondrocytes that was correlated with increased aggrecan and type II collagen protein levels [42,43]. In keeping with this finding, an ACL transection (ACLT) rat model showed reduced OARSI score after subcutaneous administration of liraglutide 50 ​μg/kg/day for 3 or 6 weeks [35].

Furthermore, the surface of cartilage is covered with a layer of phospholipids that serves as a boundary lubricant during joint loading. Any modifications to the composition of this layer could negatively affect the joint and participate in the onset of OA [44].

G protein-coupled receptor 120 (GPR-120) is a receptor for the essential omega-3-fatty acid (FA) alpha-linolenic acid. In the intestine, GPR-120 is involved in the secretion of GLP-1 upon stimulation by FAs [45]. GPR-120 is expressed by chondrocytes in culture [46], and its role in OA was investigated by inducing the ACLT post-traumatic OA model in GPR-120–knockout mice. OA-induced GPR-120-knockout mice showed accelerated OA progression as compared with OA wild-type mice. This situation translated into an increased OARSI score, increased secretion of TNF-α, increased expression of MMP-13 and COLX and bone marrow lesions [47]. In a recent study, agonist stimulation of GPR-120 in the IL-1β–stimulated ATDC5 chondrocyte cell line led to SOX9-mediated expression of type II collagen and aggrecan as well as inhibition of the expression of IL-6 and IL-8 [48]. The part of these GPR-120 chondroprotective effects due to GLP-1 secretion remains to be determined.

• **Synovial tissue and GLP-1R signalling**

Specialized cells, fibroblast-like synoviocytes and macrophage-like synoviocytes reside in the synovial membrane, composed of intimal and subintimal layers, which are responsible for notably producing hyaluronic acid and removing metabolites and matrix degradation debris, respectively [49]. Thus, these cells play a central role in maintaining synovial fluid homeostasis. In OA, the *synovium* may undergo significant changes, even before the onset of cartilage degradation, with thickening of the synovial lining layer caused by immune cell infiltration and the production of inflammatory cytokines [50]. Resident as well as infiltrated macrophages can be subject to macrophage polarization and adopt an M1 pro-inflammatory phenotype associated with increased inflammatory signalling or an M2 anti-inflammatory phenotype associated with the resolution of the inflammatory process by producing anti-inflammatory cytokines.

GLP-1R is expressed in human monocyte-derived macrophages and in the murine macrophage cell line RAW264.7 [51,52]. To date, few studies have investigated GLP-1/GLP-1R signalling in macrophages. GLP-1R activation participates in macrophage polarization by fine-tuning the level of c-Jun N-terminal kinase (JNK) versus STAT3 phosphorylation in the cell. Indeed, activation of GLP-1R triggers PKA/CREB signalling, which in turn prevents phosphorylation of JNK [52], in parallel, STAT3 phosphorylation increases. This signalling pathway, previously described in the regulation of immune responses in murine macrophage models, is essential for macrophage differentiation toward the M2 phenotype [53,54]. Placed in the context of the inflamed *synovium*, it is of importance that GLP-1–stimulated macrophages operate an M1 to M2 switch because this results in reduced IL-6, TNF-α and iNOS mRNA expression.

Therefore, GLP-1-based therapies may have protective effects by decreasing macrophage infiltration in lesions *via* inhibiting the expression of adhesion molecules [33]. For example, liraglutide can inhibit oxidized low-density lipoprotein-induced lipid accumulation and oxidative stress (ER stress) in macrophages, and this effect depends on the GLP-1R pathway [55].

The effect of GLP-1R agonists was also studied in another disease context characterized by chronic inflammation of the *synovium* and joint destruction, namely rheumatoid arthritis. In fibroblast-like rheumatoid arthritis synoviocytes, treatment with lixisenatide inhibited the inflammatory response by downregulating pro-inflammatory cytokines such as TNF, IL-6, and IL-8; inhibiting MMPs; and blunting cellular signalling pathways, including JNK, activator protein 1, and NF-κB pathways [56]. These findings confirm that GLP-1R expression extends to both cell types of the *synovium*: macrophages and fibroblast-like synoviocytes, so GLP-1R is a whole-joint tissue target for treating OA.

• **Bone and GLP-1R signalling**

Bone is a tissue with an apparent static structure but is truly dynamic and in constant remodelling throughout life, with bone resorption (mediated by osteoclasts) and bone formation (mediated by osteoblasts) occurring concomitantly [57]. During OA, subchondral bone thickens and osteophytes form. Some investigations emphasised the role of subchondral bone as an important driver of cartilage damage (disease progression), and at the cellular level, bone cells can also affect cartilage metabolism [58]. The interactions between cartilage, subchondral bone and *synovium* supports a functional link underlining strong interactions, by areas of direct contact [59] or by biochemical interactions supposed to be mainly achieved by vascularisation (oxygen and nutrient supply) [60]. Osteophytes, bone spurs growing on arthritic joints, are directly related to OA pain symptoms [61]. Several studies indicate that GLP-1 affects bone homeostasis with a proposed gut–bone axis [62]. GLP-1R is expressed in bone marrow stem cells (BMSCs) [63], osteoblasts, osteocytes [64] and osteoclasts [46], which suggests that it could play a role in both bone tissue destruction and renewal. In osteoblasts, treatment with GLP-1 and GIP incretins inhibited overexpression of the pro-degradative enzymes MMP-3 and MMP-13 in response to IL-1β stimulation [65]. *In vitro*, GLP-1, *via* c-Fos activation, intervenes in osteoblast survival and differentiation [66]. The GLP-1 analogue exendin-4 also increases proliferation, differentiation and mineralization *via* activation of the MAPK pathway [62] and β-catenin signalling [67] which act centrally in bone formation, repair and maintenance of homeostasis. Indeed, BMSCs exposed to exendin-4 showed increased gene expression of the bone transcription factors *Runx* and *Osterix* and bone matrix-producing genes *Balp* and *Bglap*. Furthermore, activation of GLP-1R in BMSCs resulted in accumulation of nuclear β-catenin, which upon association with TCF7L12 activated osteogenic genes [67].

Pereira and colleagues reported the opposite effects of GLP-1 analogues on osteoclasts and detailed the inhibitory effect of exendin-4 on osteoclast formation and bone resorption by inhibiting TNF expression in these cells [68]. Moreover, Pereira et al. showed that liraglutide and exendin-4 could increase osteoclast number but reduce the bone resorbed area per osteoclast *in vivo* [68].

Of note, receptor activator of NF-κB ligand (RANKL) is an essential cytokine that binds to the RANK receptor and is required for the formation, maturation and differentiation of osteoclasts [69]; osteoprotegerin (OPG) binds to RANKL to prevent its pro-osteoclastogenesis action. Thus, bone remodelling is fine-tuned by the ratio of OPG to RANKL levels. During OA, inflammation and production of advanced glycation end products can lead to increased RANKL signalling. In an osteoporosis context, administration of GLP-1R agonists, particularly exendin-4, reduced glucose, triglycerides, and total cholesterol levels in plasma [70] but increased osteocalcin gene expression and ratio of OPG to RANKL levels, all in favour of a beneficial role of GLP-1 in bone formation and protection [70]. These studies suggest that this peptide family might be beneficial as drugs against osteoporosis and other bone disorders, particularly in T2DM patients.

*In vivo* evidence of the role of GLP-1/GLP-1R is from *Glp-1r*−/− mice, which exhibited an increased number of osteoclasts accompanied by greater bone resorption activity and increased expression of osteoclast-related genes. These findings favour a role for GLP-1 in bone construction and preventing degradation [62]. In a recent study, a poly–GLP-1 molecule elevated TGF-β1 level in bone marrow, which resulted in increased migration of BMSCs to the bone surface. In addition, poly–GLP-1 treatment promoted BMSC differentiation to osteocytes to the detriment of adipocytes and also induced anti-inflammatory M2 polarization of bone marrow-derived macrophages [63].

• **Adipose tissue and GLP-1R signalling**

Intra-articular adipose tissue deposits, known as articular fat pads, are present in the joint, and the main adipose structure within the knee joint is the infrapatellar fat pad (IFP), also called Hoffa's fat pad. The IFP is found at the anterior front of the knee joint, between the joint capsule and the synovial membrane[71]. The IFP consists of adipocytes and is highly innervated and vascularized. Infiltration of immune cells can result in IFP inflammation. The IFP is also a source of adipokines, FAs, and FA-derived lipid mediators that could contribute to and fuel OA development in the knee joint [72,73]; obesity-associated oxidative stress induces lipolysis of adipocytes and thus increases the circulating levels of free FAs. Altered synovial fluid lipid composition has also been linked to OA [74].

Although no studies have investigated the expression of GLP-1/GLP-1R in Hoffa's tissue, several studies focused on the role of GLP-1 in white or brown adipose tissue [75,76]. Challa and colleagues showed that increased concentrations of GLP-1 or its analogue liraglutide stimulated 3T3-L1 pre-adipocyte differentiation *in vitro*, and injection of 100 ​μg/kg liraglutide twice daily for 17 days in mice fed a high fat diet for 6 weeks increased adipogenesis as compared with vehicle-treated mice [75]. Furthermore, the suppression of GLP-1R expression reduced proliferation and differentiation but induced apoptosis of pre-adipocytes by modulating the PKC signalling pathway [75]. Intriguingly, this pro-adipogenic effect of GLP-1 is accompanied by decreased body weight in liraglutide-treated mice, in keeping with the well-described effect of liraglutide [75].

However, in human mesenchymal stem cells, GLP-1 inhibited early adipocyte differentiation and instead promoted proliferation of undifferentiated mesenchymal stem cells [77]. Thus, depending on the tissue environment, cell type and cell differentiation stage, GLP-1 may exert both pro- or anti-adipogenic activity.

Murine adipose tissue macrophages express many genes characteristic of M2 macrophages, which protect adipocytes against inflammation; however, diet-induced obesity led to a shift in the activation state to an M1 pro-inflammatory state that contributes to insulin resistance [78,79]. Thus, macrophage polarization likely plays an important role in the function of adipocytes. By modulating the M1 to M2 phenotype switch, signals such as GLP-1 could indirectly safeguard adipose tissues against inflammation. Furthermore, in 3T3-Ll adipocytes, GLP-1 stimulation improved insulin responsiveness by promoting increased glucose uptake and FA synthesis in adipose cells [80], possibly by CREB phosphorylation and ERK1/2 activation [81].

Whether GLP-1R is expressed in IFP adipocytes remains to be explored, but in this eventuality, GLP-1 analogues through anti-inflammatory effects could provide a whole-joint tissue response to OA by also targeting IFP inflammation.

• **Nerves and GLP-1R signalling**

Pain is the principal reason patients seek treatment [82]. OA pain results from peripheral and central modifications in pain pathways. The cartilage itself is not innervated, but nociceptive fibers innervate the *synovium* and joint capsule, subchondral bone and *periosteum, meniscus* and ligaments [83]. The correlation between structural damage and symptoms is not established and thus constitutes a major challenge for the development of potential DMOADs [84].

In a monoiodoacetate (MIA) mouse model of OA inflammatory pain, injection of a GLP-1 analogue ameliorated pain sensitivity [85]. Although this first reported analgesic effect of a GLP-1 analogue was likely mediated by its local anti-inflammatory action, GLP-1R is also expressed in dorsal root ganglion (DRG) neurons [86] and therefore could directly act on nociceptive pain in OA DRG neurons endings are present in *synovium* [87].

The effect of GLP-1 analogues in models of neuropathy has been studied [88]. GLP-1R is expressed in microglial cells of the dorsal vertebral horn and overexpressed in models of peripheral neuropathy. Intrathecal injection of exenatide reduced the induced hypersensitivity by up to 90% in a model of peripheral neuropathy without affecting acute nociceptive responses. In addition, exenatide caused the release of β-endorphin from the spinal cord. This anti-allodynic effect induced by GLP-1R could be blocked by the opioid receptor antagonist naloxone [88].

An analgesic effect of exendin-4 was demonstrated in a rat chronic pain model of spinal nerve ligation. Intrathecal injection of exendin-4 reduced pain sensitivity as assessed by the von Frey test and decreased neuroinflammation, with reduced TNF, IL-1β and IL-6 levels, thus demonstrating that analgesic effects of GLP-1R signalling were directly correlated with its anti-neuroinflammation activity [89].

We have evidence that GLP-1 affects the nervous system, including anti-apoptotic properties in rat hippocampal neurons [90]. GLP-1R is detected in peripheral nerves of rats and mice as well as in nerve terminals of the portal vein [91]. Despite no clear demonstration of GLP-1R expression in the joint nerves, these various studies demonstrate that GLP-1R presence seems to be a possibility and that its action is real. Moreover, GLP-1R agonists significantly increased phosphorylated ERK1/2 levels in sciatic nerves from diabetic rats, so GLP-1 analogues may have selective neurotrophic and neuroprotective effects in this tissue and this localization would relieve pain in an OA context [92].

Additionally, neurogenesis may play a role in the OA pathophysiology [93]. One study demonstrated that cholinergic fibers, which play a major anti-inflammatory role in many diseases *via* acetylcholine (ACh) release after vagus nerve stimulation, are present in human OA subchondral bone [93]. This is of particular interest because *in vitro*, intestinal enteroendocrine L-cells could release GLP-1 on activation of the *α*7nACh receptor [94]. Thus, ACh release in the subchondral bone may stimulate GLP-1 release by osteoblasts or osteocytes. This remains a hypothesis and one would have to confirm both the expression of the Ach receptor on bone cells and whether GLP-1 is indeed released by these cells.

Finally, GLP-1 analogues have a long-acting effect and can pass the blood–brain barrier, which probably extends the therapeutic efficacy of GLP-1R activation.

In order to improve current knowledge on GLP-1 effects in OA, we propose a research agenda (Table 3) [[95], [96], [97], [98], [99]].

**Table 3:** Remained research questions.

**Remained research questions**
Role of GLP-1 on anabolism ? Role of GLP-1 on chondrocyte terminal differentiation ?
Role of GLP-1 on autophagy ? (95,96) Role of GLP-1: senolytic/ senomorphic ? (97,98)
Role of GLP-1 on angiogenesis ? Role of GLP-1 on subchondral bone ?
GLP-1R expression in Hoffa tissue ? Link between autophagy and adipose tissue (99) Role of GLP-1 on adipocytes ?
GLP-1R expression in neuronal cells ? AchR expression in neuronal cells ? Role of GLP-1R on neuronal apoptosis ?

## 2 Conclusion

The search for new therapeutic targets to treat people suffering from OA remains urgent as there is currently no disease-modifying therapy available for this disease. The recent discovery of anti-inflammatory, immunoregulatory and differentiation properties at the joint tissue and cellular levels of GLP-1 analogues raises the hypothesis of their potential interest to treat OA.

## Funding

Funding This research did not receive any specific grant from funding agencies in the public, commercial, or not-for-profit sectors, and no material support of any kind was received.

## References

[1] Xia B., Chen Di, Zhang J., Hu S., Jin H., Tong P. (2014). Osteoarthritis pathogenesis: a review of molecular mechanisms. Calcif Tissue Int.

[2] James S.L., Abate D., Abate K.H., Abay S.M., Abbafati C., Abbasi N. (2018). Global, regional, and national incidence, prevalence, and years lived with disability for 354 diseases and injuries for 195 countries and territories, 1990–2017: a systematic analysis for the Global Burden of Disease Study 2017. Lancet.

[3] Osteoarthritis: Structural endpoints for the development of drugs, devices, and biological products for treatment guidance for industry n.d.

[4] Kornaat P.R., Sharma R., van der Geest R.J., Lamb H.J., Kloppenburg M., Hellio le Graverand M.-P. (2009). Positive association between increased popliteal artery vessel wall thickness and generalized osteoarthritis: is OA also part of the metabolic syndrome?. Skeletal Radiol.

[5] Courties A., Sellam J., Berenbaum F. (2017). Metabolic syndrome-associated osteoarthritis. Curr Opin Rheumatol.

[6] Carbone A., Rodeo S. (2017). Review of current understanding of post-traumatic osteoarthritis resulting from sports injuries: review OF understanding OF PTOA from sports injuries. J Orthop Res.

[7] Berenbaum F. (2013). Osteoarthritis as an inflammatory disease (osteoarthritis is not osteoarthrosis!). Osteoarthritis Cartilage.

[8] Piva S.R., Susko A.M., Khoja S.S., Josbeno D.A., Fitzgerald G.K., Toledo F.G.S. (2015). Links between osteoarthritis and diabetes. Clin Geriatr Med.

[9] Berenbaum F. (2011). Diabetes-induced osteoarthritis: from a new paradigm to a new phenotype. Ann Rheum Dis.

[10] Nauck M. (2016). Incretin therapies: highlighting common features and differences in the modes of action of glucagon-like peptide-1 receptor agonists and dipeptidyl peptidase-4 inhibitors. Diabetes Obes Metabol.

[11] Gilbert M.P., Pratley R.E. (2020). GLP-1 analogs and DPP-4 inhibitors in type 2 diabetes therapy: review of head-to-head clinical trials. Front Endocrinol.

[12] Hunter D.J. (2011). Pharmacologic therapy for osteoarthritis—the era of disease modification. Nat Rev Rheumatol.

[13] Yoon G., Kim Y.-K., Song J. (2020). Glucagon-like peptide-1 suppresses neuroinflammation and improves neural structure. Pharmacol Res.

[14] Li J., Zheng J., Wang S., Lau H.K., Fathi A., Wang Q. (2017). Cardiovascular benefits of native GLP-1 and its metabolites: an indicator for GLP-1-therapy strategies. Front Physiol.

[15] Fandiño J., Toba L., González-Matías L.C., Diz-Chaves Y., Mallo F. (2020). GLP-1 receptor agonist ameliorates experimental lung fibrosis. Sci Rep.

[16] Kim S., Jeong J., Jung H.-S., Kim B., Kim Y.-E., Lim D.-S. (2017). Anti-inflammatory effect of glucagon like peptide-1 receptor agonist, exendin-4, through modulation of IB1/JIP1 expression and JNK signaling in stroke. Exp Neurobiol.

[17] Meurot C., Martin C., Sudre L., Breton J., Bougault C., Rattenbach R. (2022). Liraglutide, a glucagon-like peptide 1 receptor agonist, exerts analgesic, anti-inflammatory and anti-degradative actions in osteoarthritis. Sci Rep.

[18] La Barre Jean (1936).

[19] Graaf C de, Donnelly D., Wootten D., Lau J., Sexton P.M., Miller L.J. (2016). Glucagon-like peptide-1 and its class B G protein–coupled receptors: a long march to therapeutic successes. Pharmacol Rev.

[20] Seino Y., Fukushima M., Yabe D. (2010). GIP and GLP-1, the two incretin hormones: similarities and differences: similarities and differences of GIP and GLP-1. J Diab investig.

[21] Seino Y. (2011). Understanding the incretin effect. J Clin Endocrinol Metabol.

[22] Brown J.C., Dryburgh J.R. (1971). A gastric inhibitory polypeptide II: the complete amino acid sequence. Can J Biochem.

[23] Drucker D.J., Habener J.F., Holst J.J. (2017). Discovery, characterization, and clinical development of the glucagon-like peptides. J Clin Invest.

[24] Müller T.D., Finan B., Bloom S.R., D'Alessio D., Drucker D.J., Flatt P.R. (2019). Glucagon-like peptide 1 (GLP-1). Mol Metabol.

[25] López de Maturana R., Willshaw A., Kuntzsch A., Rudolph R., Donnelly D. (2003). The isolated N-terminal domain of the glucagon-like peptide-1 (GLP-1) receptor binds exendin peptides with much higher affinity than GLP-1. J Biol Chem.

[26] Escalada F.J. (2014). Fisiología del GLP-1 y su papel en la fisiopatología de la diabetes mellitus tipo 2. Med Clínica.

[27] Reed J., Bain S., Kanamarlapudi V. (2020). Recent advances in understanding the role of glucagon-like peptide 1. F1000Res.

[28] Berg J.K., Shenouda S.K., Heilmann C.R., Gray A.L., Holcombe J.H. (2011). Effects of exenatide twice daily versus sitagliptin on 24-h glucose, glucoregulatory and hormonal measures: a randomized, double-blind, crossover study. Diabetes Obes Metabol.

[29] Kanoski S.E., Hayes M.R., Skibicka K.P. (2016). GLP-1 and weight loss: unraveling the diverse neural circuitry. Am J Physiol Regul Integr Comp Physiol.

[30] Parthsarathy V., Hölscher C. (2013). The type 2 diabetes drug liraglutide reduces chronic inflammation induced by irradiation in the mouse brain. Eur J Pharmacol.

[31] Chaudhuri A., Ghanim H., Vora M., Sia C.L., Korzeniewski K., Dhindsa S. (2012). Exenatide exerts a potent antiinflammatory effect. J Clin Endocrinol Metabol.

[32] Iwai T., Ito S., Tanimitsu K., Udagawa S., Oka J.-I. (2006). Glucagon-like peptide-1 inhibits LPS-induced IL-1β production in cultured rat astrocytes. Neurosci Res.

[33] Lee Y.-S., Jun H.-S. (2016). Anti-inflammatory effects of GLP-1-based therapies beyond glucose control. Mediat Inflamm.

[34] Gupta S., Hawker G.A., Laporte A., Croxford R., Coyte P.C. (2005). The economic burden of disabling hip and knee osteoarthritis (OA) from the perspective of individuals living with this condition. Rheumatology.

[35] Chen J., Xie J.-J., Shi K.-S., Gu Y.-T., Wu C.-C., Xuan J. (2018). Glucagon-like peptide-1 receptor regulates endoplasmic reticulum stress-induced apoptosis and the associated inflammatory response in chondrocytes and the progression of osteoarthritis in rat. Cell Death Dis.

[36] Que Q., Guo X., Zhan L., Chen S., Zhang Z., Ni X. (2019). The GLP-1 agonist, liraglutide, ameliorates inflammation through the activation of the PKA/CREB pathway in a rat model of knee osteoarthritis. J Inflamm.

[37] Helmstädter J., Frenis K., Filippou K., Grill A., Dib M., Kalinovic S. (2020). Endothelial GLP-1 (Glucagon-Like peptide-1) receptor mediates cardiovascular protection by liraglutide in mice with experimental arterial hypertension. Arterioscler Thromb Vasc Biol.

[38] Hölscher C. (2021). Protective properties of GLP-1 and associated peptide hormones in neurodegenerative disorders. Br J Pharmacol.

[39] von Scholten B.J., Hansen T.W., Goetze J.P., Persson F., Rossing P. (2015). Glucagon-like peptide 1 receptor agonist (GLP-1 RA): long-term effect on kidney function in patients with type 2 diabetes. J Diabetes Complicat.

[40] Tanaka T., Higashijima Y., Wada T., Nangaku M. (2014). The potential for renoprotection with incretin-based drugs. Kidney Int.

[41] Loeser R.F., Goldring S.R., Scanzello C.R., Goldring M.B. (2012). Osteoarthritis: a disease of the joint as an organ. Arthritis Rheum.

[42] Mei J., Sun J., Wu J., Zheng X. (2019). Liraglutide suppresses TNF-α-induced degradation of extracellular matrix in human chondrocytes: a therapeutic implication in osteoarthritis. Am J Transl Res.

[43] Berenbaum F., Meurot C., Breton J., Sudre L., Bougault C., Rattenbach R. (2020). THU0055. Anti-degradative and pro-chondrogenic properties of liraglutide, a glucagon-like peptide 1 receptor agonist : evidence from preclinical studies and implication for osteoarthritis. Ann Rheum Dis.

[44] Kosinska M.K., Ludwig T.E., Liebisch G., Zhang R., Siebert H.-C., Wilhelm J. (2015). Articular joint lubricants during osteoarthritis and rheumatoid arthritis display altered levels and molecular species. PLoS One.

[45] Hirasawa A., Tsumaya K., Awaji T., Katsuma S., Adachi T., Yamada M. (2005). Free fatty acids regulate gut incretin glucagon-like peptide-1 secretion through GPR120. Nat Med.

[46] Koren N., Simsa-Maziel S., Shahar R., Schwartz B., Monsonego-Ornan E. (2014). Exposure to omega-3 fatty acids at early age accelerate bone growth and improve bone quality. J Nutr Biochem.

[47] Chen Y., Zhang D., Ho K.W., Lin S., Suen W.C.-W., Zhang H. (2018). GPR120 is an important inflammatory regulator in the development of osteoarthritis. Arthritis Res Ther.

[48] Xu Z., Ke T., Zhang Y., Fu C., He W. (2020). Agonism of GPR120 prevented IL-1β-induced reduction of extracellular matrix through SOX-9. Aging.

[49] Sellam J., Berenbaum F. (2010). The role of synovitis in pathophysiology and clinical symptoms of osteoarthritis. Nat Rev Rheumatol.

[50] Culemann S., Grüneboom A., Nicolás-Ávila J.Á., Weidner D., Lämmle K.F., Rothe T. (2019). Locally renewing resident synovial macrophages provide a protective barrier for the joint. Nature.

[51] Shiraishi D., Fujiwara Y., Komohara Y., Mizuta H., Takeya M. (2012). Glucagon-like peptide-1 (GLP-1) induces M2 polarization of human macrophages via STAT3 activation. Biochem Biophys Res Commun.

[52] Wan S., Sun H. (2019). Glucagon-like peptide-1 modulates RAW264.7 macrophage polarization by interfering with the JNK/STAT3 signaling pathway. Exp Ther Med.

[53] Porcheray F., Viaud S., Rimaniol A.-C., Leone C., Samah B., Dereuddre-Bosquet N. (2005). Macrophage activation switching: an asset for the resolution of inflammation. Clin Exp Immunol.

[54] Matsukawa A., Takeda K., Kudo S., Maeda T., Kagayama M., Akira S. (2003). Aberrant inflammation and lethality to septic peritonitis in mice lacking STAT3 in macrophages and neutrophils. J Immunol.

[55] Wang Y.-G., Yang T.-L. (2015). Liraglutide reduces oxidized LDL-induced oxidative stress and fatty degeneration in Raw 264.7 cells involving the AMPK/SREBP1 pathway. J Geriatr Cardiol.

[56] Du X., Zhang H., Zhang W., Wang Q., Wang W., Ge G. (2019). The protective effects of lixisenatide against inflammatory response in human rheumatoid arthritis fibroblast-like synoviocytes. Int Immunopharm.

[57] Datta H.K., Ng W.F., Walker J.A., Tuck S.P., Varanasi S.S. (2008). The cell biology of bone metabolism. J Clin Pathol.

[58] Conaghan P.G. (2005). Is progressive osteoarthritis an atheromatous vascular disease?. Ann Rheum Dis.

[59] Lyons T.J., McClure S.F., Stoddart R.W., McClure J. (2006). The normal human chondro-osseous junctional region: evidence for contact of uncalcified cartilage with subchondral bone and marrow spaces. BMC Muscoskel Disord.

[60] Malinin T., Ouellette E.A. (2000). Articular cartilage nutrition is mediated by subchondral bone: a long-term autograft study in baboons. Osteoarthritis Cartilage.

[61] Roelofs A.J., Kania K., Rafipay A.J., Sambale M., Kuwahara S.T., Collins F.L. (2020). Identification of the skeletal progenitor cells forming osteophytes in osteoarthritis. Ann Rheum Dis.

[62] Feng Y., Su L., Zhong X., Guohong W., Xiao H., Li Y. (2016). Exendin-4 promotes proliferation and differentiation of MC3T3-E1 osteoblasts by MAPKs activation. J Mol Endocrinol.

[63] Wang N., Liu X., Shi L., Liu Y., Guo S., Liu W. (2020). Identification of a prolonged action molecular GLP-1R agonist for the treatment of femoral defects. Biomater Sci.

[64] Pacheco-Pantoja E.L., Ranganath L.R., Gallagher J.A., Wilson P.J., Fraser W.D. (2011). Receptors and effects of gut hormones in three osteoblastic cell lines. BMC Physiol.

[65] Francis Berenbaum, Carole Bougault, Claire Attali (2015). https://patents.google.com/patent/EP2890390A2/en.EP2890390A2.

[66] Pacheco-Pantoja E.L., Dillon J.P., Wilson P.J.M., Fraser W.D., Gallagher J.A. (2016). c-Fos induction by gut hormones and extracellular ATP in osteoblastic-like cell lines. Purinergic Signal.

[67] Meng J., Ma X., Wang N., Jia M., Bi L., Wang Y. (2016). Activation of GLP-1 receptor promotes bone marrow stromal cell osteogenic differentiation through β-catenin. Stem Cell Reports.

[68] Pereira M., Jeyabalan J., Jørgensen C.S., Hopkinson M., Al-Jazzar A., Roux J.P. (2015). Chronic administration of Glucagon-like peptide-1 receptor agonists improves trabecular bone mass and architecture in ovariectomised mice. Bone.

[69] Mohsin S., Baniyas M.M., AlDarmaki R.S., Tekes K., Kalász H., Adeghate E.A. (2019). An update on therapies for the treatment of diabetes-induced osteoporosis. Expet Opin Biol Ther.

[70] Nuche-Berenguer B., Lozano D., Gutiérrez-Rojas I., Moreno P., Mariñoso M.L., Esbrit P. (2011). GLP-1 and exendin-4 can reverse hyperlipidic-related osteopenia. J Endocrinol.

[71] Labusca L., Zugun-Eloae F. (2018). The unexplored role of intra-articular adipose tissue in the homeostasis and pathology of articular joints. Front Vet Sci.

[72] Mustonen A.-M., Käkelä R., Lehenkari P., Huhtakangas J., Turunen S., Joukainen A. (2019). Distinct fatty acid signatures in infrapatellar fat pad and synovial fluid of patients with osteoarthritis versus rheumatoid arthritis. Arthritis Res Ther.

[73] Eymard F., Chevalier X. (2016). Inflammation of the infrapatellar fat pad. Joint Bone Spine.

[74] Van de Vyver A., Clockaerts S., van de Lest C.H.A., Wei W., Verhaar J., Van Osch G.J.V.M. (2020). Synovial fluid fatty acid profiles differ between osteoarthritis and healthy patients. CARTILAGE.

[75] Challa T.D., Beaton N., Arnold M., Rudofsky G., Langhans W., Wolfrum C. (2012). Regulation of adipocyte formation by GLP-1/GLP-1R signaling. J Biol Chem.

[76] Beiroa D., Imbernon M., Gallego R., Senra A., Herranz D., Villarroya F. (2014). GLP-1 agonism stimulates Brown adipose tissue thermogenesis and browning through hypothalamic AMPK. Diabetes.

[77] Sanz C., Vázquez P., Blázquez C., Barrio P.A., Alvarez M.D.M., Blázquez E. (2010). Signaling and biological effects of glucagon-like peptide 1 on the differentiation of mesenchymal stem cells from human bone marrow. Am J Physiol Endocrinol Metabol.

[78] Lumeng C.N., Bodzin J.L., Saltiel A.R. (2007). Obesity induces a phenotypic switch in adipose tissue macrophage polarization. J Clin Invest.

[79] Fujisaka S., Usui I., Bukhari A., Ikutani M., Oya T., Kanatani Y. (2009). Regulatory mechanisms for adipose tissue M1 and M2 macrophages in diet-induced obese mice. Diabetes.

[80] Egan J.M., Montrose-Rafizadeh C., Wang Y., Bernier M., Roth J. (1994). Glucagon-like peptide-1(7-36) amide (GLP-1) enhances insulin-stimulated glucose metabolism in 3T3-L1 adipocytes: one of several potential extrapancreatic sites of GLP-1 action. Endocrinology.

[81] Chen J., Zhao H., Ma X., Zhang Y., Lu S., Wang Y. (2017). GLP-1/GLP-1R signaling in regulation of adipocyte differentiation and lipogenesis. Cell Physiol Biochem.

[82] Paskins Z., Sanders T., Hassell A.B. (2013). What influences patients with Osteoarthritis to consult their GP about their symptoms? A narrative review. BMC Fam Pract.

[83] Syx D., Tran P.B., Miller R.E., Malfait A.-M. (2018). Peripheral mechanisms contributing to osteoarthritis pain. Curr Rheumatol Rep.

[84] Vincent T.L. (2020). Peripheral pain mechanisms in osteoarthritis. Pain.

[85] Berenbaum F., Meurot C., Vieubled M., Sudre L., Bougault C., Rattenbach R. (2020). Protective effects of intra-articular formulated liraglutide in osteoarthritis : preclinical studies. Osteoarthritis Cartilage.

[86] Neogi T., Guermazi A., Roemer F., Nevitt M.C., Scholz J., Arendt-Nielsen L. (2016). Association of joint inflammation with pain sensitization in knee osteoarthritis: the multicenter osteoarthritis study: MRI lesions and sensitization IN knee OA. Arthritis Rheumatol.

[87] Richter F., Natura G., Löser S., Schmidt K., Viisanen H., Schaible H.-G. (2010). Tumor necrosis factor causes persistent sensitization of joint nociceptors to mechanical stimuli in rats: sensitization of Joint Nociceptors by TNF. Arthritis Rheum.

[88] Gong N., Xiao Q., Zhu B., Zhang C.-Y., Wang Y.-C., Fan H. (2014). Activation of spinal glucagon-like peptide-1 receptors specifically suppresses pain hypersensitivity. J Neurosci.

[89] Cui S.-S., Feng X.-B., Zhang B.-H., Xia Z.-Y., Zhan L.-Y. (2020). Exendin-4 attenuates pain-induced cognitive impairment by alleviating hippocampal neuroinflammation in a rat model of spinal nerve ligation. Neural Regen Res.

[90] Perry T., Haughey N.J., Mattson M.P., Egan J.M., Greig N.H. (2002). Protection and reversal of excitotoxic neuronal damage by glucagon-like peptide-1 and exendin-4. J Pharmacol Exp Therapeut.

[91] Vahl T.P., Tauchi M., Durler T.S., Elfers E.E., Fernandes T.M., Bitner R.D. (2007). Glucagon-like peptide-1 (GLP-1) receptors expressed on nerve terminals in the portal vein mediate the effects of endogenous GLP-1 on glucose tolerance in rats. Endocrinology.

[92] Jolivalt C.G., Fineman M., Deacon C.F., Carr R.D., Calcutt N.A. (2011). GLP-1 signals via ERK in peripheral nerve and prevents nerve dysfunction in diabetic mice. Diabetes Obes Metabol.

[93] Courties A., Belle M., Senay S., Cambon-Binder A., Sautet A., Chédotal A. (2020). Clearing method for 3-dimensional immunofluorescence of osteoarthritic subchondral human bone reveals peripheral cholinergic nerves. Sci Rep.

[94] Wang D., Meng Q., Leech C.A., Yepuri N., Zhang L., Holz G.G. (2018). α7 nicotinic acetylcholine receptor regulates the function and viability of L cells. Endocrinology.

[95] Zhang Q., Liu Q., Niu C.Y. (2021). [Liraglutide alleviates lipotoxic liver cell damage and promotes autophagy to improve non-alcoholic fatty liver]. Zhonghua Gan Zang Bing Za Zhi.

[96] Duan R., Xie H., Liu Z.-Z. (2020). The role of autophagy in osteoarthritis. Front Cell Dev Biol.

[97] Wang K., Chen X., Chen Y., Sheng S., Huang Z. (2020). Grape seed procyanidins suppress the apoptosis and senescence of chondrocytes and ameliorates osteoarthritis *via* the DPP4-Sirt1 pathway. Food Funct.

[98] McCulloch K., Litherland G.J., Rai T.S. (2017). Cellular senescence in osteoarthritis pathology. Aging Cell.

[99] Ferhat M., Funai K., Boudina S. (2019). Autophagy in adipose tissue physiology and pathophysiology. Antioxidants Redox Signal.

